# Distributed cognitive maps reflecting real distances between places and views in the human brain

**DOI:** 10.3389/fnhum.2014.00716

**Published:** 2014-09-11

**Authors:** Valentina Sulpizio, Giorgia Committeri, Gaspare Galati

**Affiliations:** ^1^Laboratory of Neuropsychology, Fondazione Santa Lucia IRCCSRoma, Italy; ^2^Department of Neuroscience, Imaging and Clinical Sciences, University G. d’Annunzio, and ITAB, Institute for Advanced Biomedical Technologies, G. d’Annunzio FoundationChieti, Italy; ^3^Department of Psychology, Sapienza UniversityRome, Italy

**Keywords:** spatial representation, navigation, hippocampus, retrosplenial complex, parahippocampal place area, multivoxel pattern analysis, representational similarity analysis, functional magnetic resonance imaging (fMRI)

## Abstract

Keeping oriented in the environment is a multifaceted ability that requires knowledge of at least three pieces of information: one’s own location (“place”) and orientation (“heading”) within the environment, and which location in the environment one is looking at (“view”). We used functional magnetic resonance imaging (fMRI) in humans to examine the neural signatures of these information. Participants were scanned while viewing snapshots which varied for place, view and heading within a virtual room. We observed adaptation effects, proportional to the physical distances between consecutive places and views, in scene-responsive (retrosplenial complex and parahippocampal gyrus), fronto-parietal and lateral occipital regions. Multivoxel pattern classification of signals in scene-responsive regions and in the hippocampus allowed supra-chance decoding of place, view and heading, and revealed the existence of map-like representations, where places and views closer in physical space entailed activity patterns more similar in neural representational space. The pattern of hippocampal activity reflected both view- and place-based distances, the pattern of parahippocampal activity preferentially discriminated between views, and the pattern of retrosplenial activity combined place and view information, while the fronto-parietal cortex only showed transient effects of changes in place, view, and heading. Our findings provide evidence for the presence of map-like spatial representations which reflect metric distances in terms of both one’s own and landmark locations.

## Introduction

Orienting in large-scale space requires an accurate representation of one’s spatial position and orientation within the environment, as well as knowledge about how portions of the environment appear from multiple views. Evidence of how these core features are implemented at the neuronal level comes from neurophysiological experiments showing striking spatial correlates of neuronal firing in freely moving animals. “Place cells” in the hippocampus fire whenever the animal is at a particular location (O’Keefe and Dostrovsky, 1971), as determined by the geometry of the environment (O’Keefe and Burgess, 1996), independently from the direction of its head and from where it is looking at. “Spatial view cells” in the primate hippocampus and parahippocampal gyrus fire when the animal looks at a specific part of an environment (Rolls, 1999), independently from its location and from its heading direction. “Head-direction cells” in Papez circuit structures, including the retrosplenial region, fire only when the animal maintains a certain heading or orientation within the environment (Chen et al., 1994; Taube, 1998), independently from its location and from where it is looking at. The existence of neurons selectively tuned to these three features represents the most direct evidence of an allocentric (world-centered) representation of the surrounding space.

In humans, the neural systems supporting allocentric spatial representations are less well understood. Neuroimaging studies on human spatial navigation and orientation have disclosed the role of ventromedial posterior cortical regions such the parahippocampal place area (PPA) and the retrosplenial complex (RSC) (reviewed in Epstein, 2008) in spatial orienting and navigation: these regions also respond during passive viewing of navigationally relevant visual stimuli such as scenes and buildings (Epstein et al., 1999; Epstein and Higgins, 2007; see also Epstein, 2008). In particular, PPA has been implicated in landmark recognition (Epstein and Vass, 2013), perception of the visuo-spatial structure of the local scene, and selective discrimination of different views (Park and Chun, 2009). Accordingly, view cells have been discovered in the human parahippocampal gyrus through intracranial recordings (Ekstrom et al., 2003). RSC is active during real and imagined navigation (Ino et al., 2002; Wolbers and Büchel, 2005), retrieval of environment-centered information (Committeri et al., 2004; Galati et al., 2010; Sulpizio et al., 2013), and in the presence of permanent items within the scene (Auger et al., 2012; Auger and Maguire, 2013). Although several imaging studies reported the involvement of the hippocampus in spatial navigation and/or map-like representations (Ghaem et al., 1997; Maguire et al., 1998; Wolbers and Büchel, 2005; Iaria et al., 2007; Wolbers et al., 2007; Baumann et al., 2010, 2012; Brown et al., 2010, 2012; Morgan et al., 2011; Viard et al., 2011; Baumann and Mattingley, 2013; Brown and Stern, 2014), the discovery of place cells in intracranial recordings in the human hippocampus represents the most direct evidence of a place representation in the human brain (Ekstrom et al., 2003).

Taken together, this evidence indicates that hippocampal, parahippocampal and retrosplenial areas are the key nodes of the neuronal network that supports representation of large-scale space in humans. But what are the specific features represented at each of these nodes? A recent imaging study (Vass and Epstein, 2013) addressed this issue by using multivariate pattern analysis to identify brain regions that encode location and heading direction within a large-scale, real-world familiar environment. Activity patterns in RSC, left presubiculum and medial parietal cortex were found to contain information about location but not about heading, which was instead represented in the right presubiculum. Morgan et al. (2011) showed that the human hippocampus encodes real-world distances between landmarks. Taken together, these pieces of evidence represent an important step toward understanding how allocentric information are represented in the human brain, but leave many questions unanswered.

For example, which brain area encodes the spatial location one is looking at independently from the one’s own location and heading direction, similarly to spatial view cells? While Vass and Epstein (2013) explored the neural code of heading direction and spatial position, our study, by using a small-scale, fully controlled, virtual reality environment, aimed at the complete disentanglement of place-, view-, and heading-related representations, similarly to what has been done in neurophysiological studies on animals (see above: Chen et al., 1994; Taube, 1998; Rolls, 1999). This allowed to study both self-location and the viewed location within the same environment. While Morgan et al. (2011) searched for a map-like, distance-related spatial representation of the observed landmarks, we aimed at identifying whether all the above mentioned representations (place-, view-, and heading-based) are sensitive to real-world distances.

We asked participants to perform an incidental go-nogo task on a series of snapshots taken from a virtual room, which varied for (1) the participant’s virtual position (place); (2) the participant’s world-centered orientation (heading); and (3) the portion of the scene located just in front of them (view). Snapshots were presented in a continuous carry-over sequence (Aguirre, 2007) while participants were scanned with functional magnetic resonance imaging (fMRI).

We dealt with a number of questions about where and how the brain automatically encodes place, heading and view during exposure to a familiar environment. Do the hippocampus, parahippocampal gyrus and retrosplenial cortex encode the allocentric location of the observer, of the observed portion of the environment, or both? Is heading also represented in the same regions? Are the three features represented topographically, with closer physical locations entailing activity patterns more similar in neural representational space? We also explored whether and how other regions contribute to the coding of these features. To answer to these questions we used a combination of univariate analysis of adaptation effects (Grill-Spector et al., 2006) induced by repetition of any of the three features across consecutive snapshots, and multivariate pattern analysis (Morgan et al., 2011; Epstein and Morgan, 2012). We found evidence of a distributed map-like spatial representation which reflects metric distances in terms of both one’s own and landmark locations within the environment.

## Materials and methods

### Subjects

Sixteen neurologically normal volunteers (eight males, mean age 27.4 yrs, s.d. 3.8) participated to the study, and underwent a behavioral familiarization and training session and then an fMRI acquisition session. All subjects were right handed, as assessed by the Edinburgh Handedness Inventory (Oldfield, 1971: mean index = 0.71, s.d. 0.21) and had normal or corrected-to-normal vision. The protocol was approved by the G. d’Annunzio University of Chieti’s institutional ethics committee and written informed consent was obtained from each participant before starting the study.

### Stimuli and task

**Figure 1:**
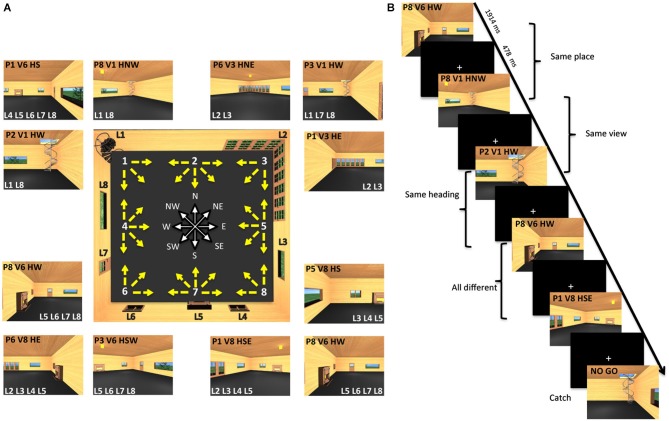
**Virtual environment and experimental paradigm. (A)** The central panel shows a map of the virtual room and all 32 pictures employed as stimuli. Each picture corresponds to a yellow arrow. The origin of the arrow corresponds to the position of the virtual camera and is defined as place (P). The direction of the arrow corresponds to the direction of the virtual camera and is defined as heading (H). The location pointed at by the arrow determines what is visible in the picture and is defined as view (V). There are eight possible places and views, marked by white numbers on the map, and eight possible heading directions, marked by the corresponding cardinal orientations. Eight different landmarks (L) are placed on the room walls. Examples of actual pictures are shown all around the map: each is identified through its place (P), view (V), and heading (H). These pictures are also characterized by the visibility of a set of landmarks (L). **(B)** Example of trial sequence. Participants were presented a series of pictures and distinguished between pictures of the familiar room (target trials) and unfamiliar pictures (catch trials). Trial stimuli are shown in which either the place or the view or the heading are the same as in the previous trial.

The virtual environment (Figure 1A, central panel) was designed using 3Dstudio Max 9 (Autodesk Inc., San Rafael, CA, USA), and represented an internal view of a living room, containing eight “landmarks”, i.e., fixed cues on the walls, including, one spiral staircase, one large French corner window, one wide window, one small grating window, one fireplace, one small grating window, one door, and one wide window (labeled as L1-L8 in Figure 1A). The room was designed with a square plan so that the four walls could be distinguished only on the basis of the layout of these distinctive cues.

Stimuli were 32 different snapshots of this virtual environment. Each snapshot simulated a photograph of the environment taken with a 24-mm lens (74 by 59° simulated field of view), which described a specific place, view and heading direction within the room. The observer’s positions (place) were arranged from 1–8 around the perimeter of the room (Figure 1A, central panel). These positions corresponded to the location of the virtual camera used to take the snapshots. Each camera could be oriented in different directions, as illustrated by the yellow arrows. The target of these cameras corresponded to one of the eight environmental points where the observer was looking at (view). The camera orientation defined the observer’s orientation (heading), which corresponded to one of the eight cardinal directions (North or N, North-East or NE, East or E, South-East or SE, South or S, South-West or SW, West or W, North-West or NW) represented in white by the wind rose. Examples of stimuli taken from a specific place, and with a specific view and heading within the room, are shown in the external panels of Figure 1A (for example, the snapshot P1V6HS depicted Place 1, View 6 and Heading South). Each example also reports the set of landmarks visible in the snapshot.

During the fMRI acquisition runs, participants viewed sequences of images taken from the virtual room, each characterized by a specific place, view and heading within the room. These images were presented in a continuous carry-over sequence (Aguirre, 2007) that counterbalanced main effects and first-order carry-over effects by ensuring that each trial was preceded by every other trial equally often. This allowed us to use the same data for both univariate (fMRI adaptation or fMRIa) and multivariate analyses (multivariate pattern analysis or MVPA) (Morgan et al., 2011; Epstein and Morgan, 2012). On each trial, participants were instructed to press a button when the presented picture was taken from the familiar room (target trials) and withhold in the case of an unfamiliar picture (catch trials) (Figure 1B). Catch trials were modified snapshots of the virtual room in which the relative position of the fixed environmental cues was altered. Note that the purpose of the task was merely to prompt participants to pay close attention to the images, but the familiarity judgment itself was independent of the specific features of each image, such as the depicted place, view, and heading and their relationship with the features depicted in the previous trials, which were the two key items on which data analysis was based (see below).

### Apparatus and procedure

Images were acquired using a 3 T Achieva Philips MR system, operating at the Institute for Advanced Biomedical Technologies (ITAB), G. d’Annunzio University, Chieti, using a standard head coil. Stimuli were generated by a control computer located outside the MR room, running in-house software (see Galati et al., 2008) implemented in MATLAB (The MathWorks Inc., Natick, MA, USA). An LCD video projector with a customized lens was used to project wide-field visual stimuli (7.5 by 36.5° of visual angle) to a back projection screen mounted inside the MR tube and visible through a mirror mounted inside the head coil. Presentation timing was controlled and triggered by the acquisition of fMRI images. Responses were given through push buttons connected to the control computer via optic fibers.

Echo-planar functional MR images (TR = 1914 ms, TE = 25 ms, flip angle = 80°, 64 × 64 image matrix, 3.6 × 3.6 mm in-plane resolution, 39 slices, 3.59 mm slice thickness with no gap, interleaved excitation order) were acquired in the AC–PC plane using blood-oxygenation level-dependent imaging (Kwong et al., 1992). The first four volumes of each scan were discarded to achieve steady-state T1 weighting, and the experimental tasks started at the beginning of the fifth image. From the superior convexity, sampling included all the cerebral cortex, excluding only the ventral portion of the cerebellum. A three-dimensional high resolution anatomical image was also acquired for each subject (Philips MPRAGE sequence, TR = 8 s, TE = 3.7 ms, flip angle = 8°, 512 × 512 image matrix, 1 × 1 mm in-plane resolution, 160 contiguous oblique slices).

Each subject first underwent a training session aimed at familiarizing the participant with the virtual environment. We presented a 52-s movie consisting of a 360° tour of the virtual room. Subjects were instructed to memorize the global spatial layout of the room until they were sure to be able to draw a sketch representing the survey perspective of the room. After familiarization, subjects completed a training task that included a series of questions about their own position (place), view and heading within the room. In each trial, participants were shown the letter P, V and H for 300 ms at the center of the screen, instructing the place, view and heading questions, respectively. Then, a picture of the room was shown from an unpredictable viewpoint for 2 s (study phase). According to the instruction, participants encoded their own place, view or heading within the room. After a short delay, participants were shown a picture (test phase) representing either the map of the room (in case of place and view questions) or the wind rose (in case of heading questions). An eight-answer choice was shown on this picture and participants selected the memorized place, view and heading within the room by pressing the corresponding button on the 8-button response device. The test picture lasted on the screen until participants answered and the following trial started after a fixed inter-trial interval (ITI, 2 s) and after feedback on the accuracy. This training phase was crucial to ensure that participants developed a long-term knowledge of the room layout and were able to encode the current place, view and heading within the room. The training sessions were repeated until participants reached a threshold accuracy of at least 70% for all three questions. Average final accuracy was 91% for heading and 84% for both place and view.

Then participants underwent an fMRI acquisition session, consisting in the main experiment and in two “localizer” imaging runs. The main experiment consisted of four fMRI runs lasting approximately 11 min (337 functional MR volumes), comprising 176 target trials and 25 catch trials, plus 10 fixation periods each lasting 7656 ms long (rest). Each stimulus was presented for 1913 ms (corresponding to 1 MR volume), followed by an inter-trial interval of 478 ms (corresponding to 1/4 MR volume). Fixation periods were 7757 ms long (corresponding to 4 MR volumes).

Localizer imaging runs were aimed at identifying scene-responsive regions in the parahippocampal and retrosplenial cortex (Epstein, 2008). In each imaging run, participants passively viewed eight alternating blocks (16 s) of photographs of faces and places/scenes presented for 300 ms every 500 ms, interleaved with fixation periods of 15 s on average (see Sulpizio et al., 2013). During each run we acquired 234 functional MR volumes, for a total of 7 min each. For some subjects localizer runs were acquired in a separate day.

### Image preprocessing and analysis

Image preprocessing and analysis was performed using a combination of different tools: SPM8 (Wellcome Department of Cognitive Neurology, London, UK) for image preprocessing, univariate analysis, functional regions of interest (ROI) definition, and ROI data extraction; AAL (Tzourio-Mazoyer et al., 2002) for anatomical ROI definition; libSVM (Chang and Lin, 2011) for classification analyses; custom scripts implemented in Matlab (The MathWorks Inc., Natick, MA, USA) for representation similarity analysis.

Functional time series from each subject were first temporally corrected for slice timing, using the middle slice acquired in time as a reference, and then spatially corrected for head movement, using a least-squares approach and six parameter rigid body spatial transformations. They were then spatially normalized using an automatic nonlinear stereotaxic normalization procedure (final voxel size: 3 × 3 × 3 mm). Data for all univariate analyses, including the functional localizer imaging runs, were spatially smoothed with a three dimensional Gaussian filter (6 mm full-width-half-maximum); data for multivoxel patterns analyses (MVPA) were not smoothed. The template image for spatial normalization was based on average data provided by the Montreal Neurological Institute (Mazziotta et al., 1995) and conforms to a standard coordinate referencing system (Talairach and Tournoux, 1988).

The time series of functional MR images obtained from each participant was analyzed separately. The effects of the experimental paradigm were estimated on a voxel-by-voxel basis, according to the general linear model (GLM) extended to allow the analysis of fMRI data as a time series. The onset of each trial constituted a neural event, that was modeled through a canonical hemodynamic response function. Separate regressors were included for each trial type, yielding parameter estimates for the average hemodynamic response evoked by each. The model included a temporal high-pass filter to remove low-frequency confounds with a period above 128 s. Serial correlation in the fMRI time series were estimated with a restricted maximum likelihood (ReML) algorithm using an autoregressive AR(1) model during parameter estimation, assuming the same correlation structure for each voxel, within each imaging run. The ReML estimates were then used to whiten the data.

**Figure 2:**
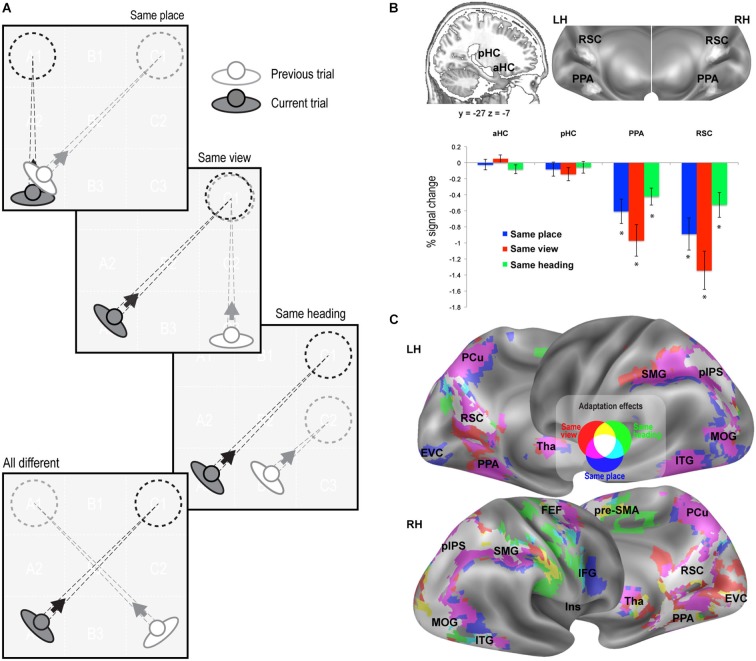
**fMR adaptation results. (A)** Schematic illustration of the rationale of the adaptation analysis. Each trial (current trial) was labeled according to its relationship with the previous trial (same place, same view, same heading, or all different). **(B)** Adaptation effects in predefined ROIs. Top: Anatomical localization of the anterior (aHC) and posterior hippocampus (pHC) for one sagittal slice (left) and superposition of individually-defined parahippocampal place area (PPA) and retrosplenial complex (RSC) on the medial/inferior view of the cortical surface of the left (LH) and right (RH) hemispheres (right). Bottom: Plots show adaptation effects for place, view, heading, i.e., reduction of estimated BOLD signal in same-place, same-view, and same-heading trials compared to all-different trials. * *p* < 0.001. **(C)** Whole-brain adaptation effects. Voxels showing significant response attenuation for same-place (blue), same-view (red), and same-heading trials (green) as compared to all-different trials, are superimposed on reconstructions of the lateral and medial surfaces of the left (LH) and right (RH) hemispheres.

**Figure 3:**
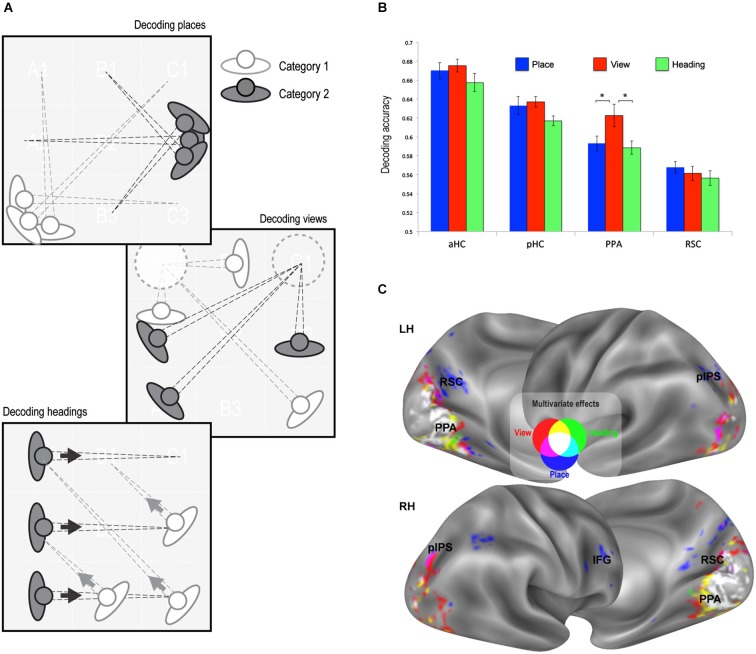
**Multivariate classification results. (A)** Schematic illustration of the rationale of the classification analysis. Each trial was labeled according to its place, view, and heading. A linear classifier was trained to decode between trials corresponding to each pair of places, views, or heading directions. **(B)** Classification accuracy in the predefined ROIs (all above chance, *p* < 0.001). Classification performance was higher for view than for place and heading only in the PPA. * *p* < 0.01. **(C)** Whole-brain classification accuracy. Voxels decoding places (blue), views (red) and heading directions (green) significantly better than chance (minimum accuracy = 0.57, *p* < 0.01 corrected) are superimposed on the lateral and medial surfaces of both left (LH) and right (RH) hemispheres.

**Figure 4:**
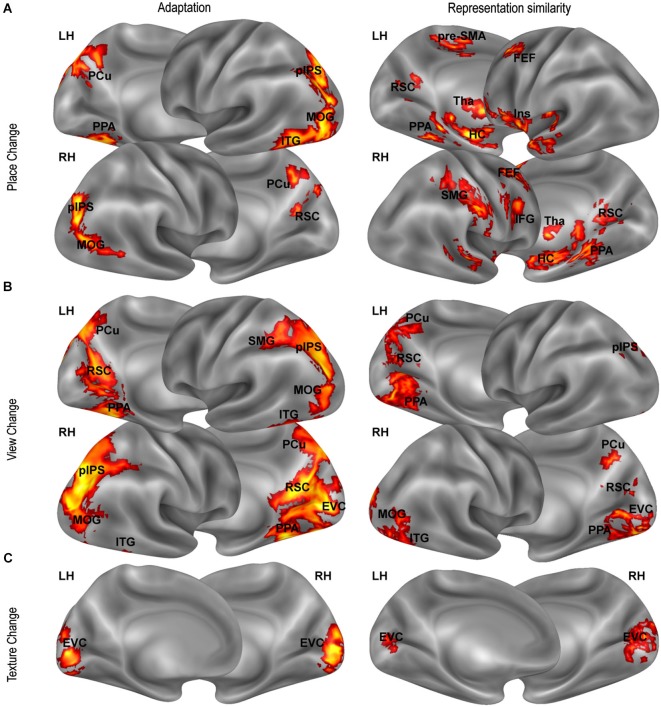
**Distance-related effects**. Distance-related effects, as assessed by fMR adaptation (left column) and representational similarity analysis (right column). Maps show univariate (left panels) and multivariate (right panels) responses that scale with **(A)** position-related (Place Change) and **(B)** view-related (View Change) distances and with **(C)** texture dissimilarity (Texture Change) .

We employed different GLMs to model different features of the images presented in each trial (see below). In all models, target trials with no response (1% on average across subjects) and catch trials were modeled as separate conditions and excluded from further analysis. For all analyses, images of parameter estimates derived from each individual GLM and representing the estimated amplitude of the hemodynamic response in each modeled condition were entered into one-sample or paired *t*-tests, to test hypotheses about the presence of an effect or about differential effects across pairs of conditions, respectively, in the whole population our participants were extracted from. For each effect of interest, we obtained a statistical parametric map of the *t*-statistic, which was thresholded at *p* < 0.05, corrected for multiple comparisons using a topological false discovery rate procedure based on random field theory (Chumbley et al., 2010). The renderings in Figures 2–4 were created by projecting thresholded statistical maps onto cortical surface reconstructions of an average brain from the Conte69 atlas (Van Essen, 2005) using an in-house Matlab toolbox (BrainShow).

All analyses were conducted both on the whole brain at the voxel level, and on three independently defined, theoretically motivated, ROIs. Two of them, the PPA and the RSC, were defined in each individual participant by analyzing “localizer” imaging runs. For localizer runs, place/scene and face blocks were modeled as box-car functions, convolved with a canonical hemodynamic response function. The PPA and the RSC were identified in individual subjects as the regions responding more strongly to places/scenes than to faces in the posterior parahippocampal cortex and in the RSC, respectively. The RSC was defined extensively, following Epstein (2008) to include the posterior cingulate (Brodmann areas 23–31), the retrosplenial cortex proper (Brodmann areas 29–30), and the nearby ventral parietal-occipital sulcus and anterior calcarine sulcus. Individual regions of interest were created by selecting all activated voxels (*p* < 0.05 corrected) at a maximum distance of 16 mm from the activation peak and used for independent time series analysis during the main experiment. The third region, the hippocampus (HC), was instead defined anatomically and was split into an anterior (aHC) and a posterior (pHC) ROI by an axial division at *z* = −9 (according to Morgan et al., 2011). This was motivated by the recent proposal (Baumann and Mattingley, 2013) that the posterior hippocampus would maintain stable and detailed spatial representations, while the anterior hippocampus would store more integrated but coarse representations, which support the flexible planning of routes. Anatomical localization, regional peaks and size of each ROI are detailed in Figure 2B and Table 1. ROI analyses were conducted by averaging preprocessed voxel time series across all voxels within each ROI, and entering the averaged regional time courses into the GLM.

**Table 1:** **Regional peaks (MNI coordinates) and size (mm^3^) of the regions of interest (ROIs)**.

Region	Hemisphere	MNI coordinates			Size (mm^3^)
		*x*	*y*	*z*	
aHC	Left	−33	−8	−28	4779
	Right	27	−6	−28	4941
pHC	Left	−32	−36	−9	2646
	Right	34	−36	−9	2646
PPA	Left	−28	−50	−8	1728
	Right	28	−50	−10	1701
RSC	Left	−18	−56	9	864
	Right	19	−56	11	1323

*aHC: anterior hippocampus; pHC: posterior hippocampus; PPA: parahippocampal place area; RSC: retrosplenial complex*.

### fMR adaptation analysis

This analysis were aimed at demonstrating the presence of a representation of places (views, headings), in a specific voxel or ROI, on the basis of the assumption that a repetition of the same place (view, heading) across two consecutive trials produces a reduction of the neural response to the second trial, as indexed by the amplitude of the event-related BOLD signal (fMR adaptation). For this purpose, we modeled each target trial on the basis of its relationship with the preceding target trial in terms of same/different place, view, and heading. This resulted in the following condition labels: (1) *same place*, for pictures taken from the same place as the previous trial, although with a different heading and thus showing a different view; (2) *same view*, for pictures showing the same view as the previous trial, although taken from a different place and thus with a different heading; (3) *same heading*, for pictures taken in a direction parallel to the previous trial, but from a different place and thus showing a different view; (4) *all same*, for pictures identical to the previous trial; and (5) *all different*, for pictures where neither the place nor the view nor the heading was the same as the previous trial. Figure 1B shows an exemplar sequence with the corresponding trial labels. Note how each trial is labeled relative to the previous one, while at the same time contributing to determine the label of the next one (carry-over sequence).

The model also included two variables as parametric modulators of the amplitude of the BOLD response, in order to control for two potential confounds. First, we explicitly modeled low-level visual dissimilarities between the current and the previous image (texture change). This index was introduced (as in Epstein and Morgan, 2012) since fMR adaptation can occur because of low-level similarities between images, irrespective of spatial differences. Texture change was computed between each pair of images through a texture model (Renninger and Malik, 2004). Briefly, images were converted to grayscale and passed with V1-like filters to generate a list of the 100 most prototypical texture features found across the images (MATLAB code available at renningerlab.org). A histogram of texture frequency was then generated for each image. The visual dissimilarity between a pair of images was calculated by comparing the distribution of the two histograms using a chi square measure (smaller chi square values correspond to more similar images).

The second confound we controlled for was the amount of similarity between the current and the previous image in terms of the set of visible landmarks. Indeed, adaptation could occur because a given region may selectively respond to the identity of the visible landmarks irrespective of their location and thus adapt to the repeated presentation of the same landmark. Thus we built an index of “landmark similarity” between images, computed as the number of shared visible landmarks between two images. For example, in the pictures labeled as P1 V6 HS (Figure 1A, upper left corner) and P6 V8 HE (Figure 1A, lower left corner) there are two shared landmarks (L4 and L5), so the landmark similarity index would be equal to two. We also tried alternative methods of defining landmark similarity, such as computing the proportion of shared landmarks relative to the total number of visible landmarks in the two images (which accounts not only for shared but also for non-shared landmarks), or simply considering whether there is at least one shared landmark or not (a binary regressor). All these measures were strongly correlated to each other, and gave nearly identical results.

Target trials with missing responses, or following a fixation period, a catch trial, or a target trial with missed response, were modeled separately and excluded from the analysis. At the group level, adaptation was defined as a significant reduction of estimated BOLD response to same-place, same-view or same-heading trials compared to all-different trials.

### Multivariate classification analysis

An alternative strategy to demonstrate the presence of a neural representation of place, view, and heading was based on a multivariate classification analysis, where a classifier was trained to discriminate multi-voxel patterns of estimated BOLD responses to pairs of places, views, or heading directions (see Norman et al., 2006 for a review). Classification outcomes significantly above chance were taken as evidence of the presence of place-, view-, or heading-related information in the analyzed region. Multivariate analyses were conducted both on patterns extracted from the predefined ROIs, and on the whole brain through the “searchlight” approach (Kriegeskorte et al., 2006), i.e., by moving a small spherical ROI (diameter 9 mm) on each voxel of the gray matter in turn, classifying the patterns extracted from that ROI, and assigning the classification outcome score to the ROI central voxel, thus creating a whole-brain image of classification success.

We first ran a GLM on unsmoothed preprocessed images of each fMRI run where each of the 32 images resulting from the possible combinations of place, view, and heading (see Figure 1A) was modeled as a different experimental condition. This yielded a voxel-by-voxel estimate (i.e., an image) of the amplitude of the BOLD response evoked by each of the 32 trial types across all repetitions of that trial type within each run. Classification was then performed on multi-voxel patterns extracted from these images, separately for places, views, and heading directions. For each of the three features, we assigned each image to one of eight categories, representing the eight possible places (P1-P8), views (V1-V8), or heading directions (H1-H8) within the virtual room (Figure 1A). Then, for each pair of categories, we trained a linear support vector machine (SVM) classifier to learn discriminate the corresponding image. A leave-one-out cross-validation procedure was used to test classification outcomes on a data set independent from that used for training the classifier: images from all runs except one were used in turn to train the classifier and the remaining data were used to evaluate prediction accuracy. The SVM was used in combination with a recursive feature elimination (RFE) procedure (De Martino et al., 2008), which helps detecting sparse discriminative patterns within a ROI by progressively discarding voxels with the lowest discriminative power. The resulting classification outcomes were averaged across cross-validation folds and category pairs. At the group level, the distributions of classification outcomes were tested against chance level, i.e., 0.5, through one-sample *t*-tests.

### Distance-related adaptation analysis

A second analysis based on fMR adaptation was performed to explore whether neural representations reflected real distances between places, views, and heading directions. We examined whether adaptation effects between pairs of images depended on the spatial differences between them. To this aim we modeled physical distances in place, view, and heading direction between the current and the previous trials as parametric modulators of the BOLD response. For this analysis, we built a GLM where all target trials with a valid response and preceded by a target trial with a valid response were modeled as trials of the same type, but allowing a polynomial modulation up to the third-order of the response amplitude by five different variables (see below). We chose a polynomial rather than a linear modulation because we could not take it for granted that a relationship between physical distances and adaptation effects would necessarily have a linear shape. All-same trials, where pictures were identical to the previous trials, were modeled separately and not considered here, as also target trials with missing responses, or following a fixation period, a catch trial, or another target trial with missed response.

Three of the considered modulatory variables modeled the three spatial quantities of interest: (1) place change, i.e., the physical distance between the position from which the current and the previous trial pictures were taken; (2) view change, i.e., the physical distance between the locations shown in the current and the previous trial pictures; and (3) heading change, i.e., the angular displacement between the allocentric directions of the current and the previous trial pictures. The other two modulatory variables (texture change and landmark similarity: see *fMR adaptation analysis* above for details) controlled for the potential confounds induced by similarity between the current and the previous trial in terms of low-level visual parameters and of the set of visible landmarks, respectively. The texture change parameter was positively correlated with view change, as expected, since images depicting the same view (i.e., the same landmark) have more chances to be perceptually similar. For this reason we orthogonalized the view change relative to the texture change parameter. Similarly, landmark similarity was negatively correlated with both view and heading changes, so both parameters were orthogonalized relative to landmark similarity.

Since heading change was positively correlated with both place change (*r* = 0.18, *p* < 0.0001) and view change (*r* = 0.39, *p* < 0.0001), we implemented two different models to test (1) distance-related adaptation effects for place and view changes; and (2) heading-related adaptation effects. At the group level, adaptation for place, view, heading, texture, and landmarks was defined as a significant positive loading of the corresponding parameter estimate (tested through a one-sample *t*-test across subjects), indicating that the BOLD response is positively modulated by change.

### Representational similarity analysis

Representational similarity analysis (Kriegeskorte et al., 2008) was used as a complement to distance-related adaptation analysis to test whether similarities between multivoxel patterns reflected physical distances (between places, views and heading directions), visual dissimilarities (in terms of differences in texture features between pairs of images), and landmark dissimilarities (in terms of the proportion of non-shared visible landmarks between pairs of images). As for the multivariate classification analysis, representational similarity analysis was conducted both on patterns extracted from the predefined ROIs, and on the whole brain through a “searchlight” approach, starting from estimates of amplitude of the BOLD response elicited by each of 32 possible combinations of place, view, and heading. For each subject and for each analyzed region, we extracted the corresponding 32 patterns and computed a neural distance matrix between each possible pair, with neural distance computed as the Euclidean distance between the multivoxel patterns. The linear relationship between neural distances and physical distances was then tested with multiple regression models, where place, view and heading “physical” distances, and low-level visual and landmark dissimilarities between the corresponding images were used as predictors.

This analysis thus aimed at determining whether dissimilarities between neural representations evoked by the different pictures could be explained by spatial differences between the displayed places, views, and heading directions, over and above low-level perceptual dissimilarities and differences in the set of visible landmarks between the images. As for the distance-related adaptation analysis, we created two different regression models for places and views and for heading directions, respectively. For each model, the average of regression parameter estimates from each subject was compared to zero using a one-tailed *t*-test. In all these regions the effects of regressors were tested against a Bonferroni-corrected significance threshold (*p* < 0.01).

### Cross-decoding between places and views

In a further analysis, we explored the existence of a neural code that generalizes across being in one location (place) and looking at that location (view). We used a cross-decoding approach to directly examine whether differences between the voxel activity patterns elicited by two different places were similar to the differences between the voxels activity patterns elicited by the two different views in the same brain area. To this aim, we trained the classifier to discriminate between sets of pictures taken from two different locations (places), and checked whether the classifier was able to discriminate between sets of pictures where those two spatial locations were the camera targets (views). We also tried the opposite way, i.e., training the classifier on stimuli categorized by view and testing its ability to classify places.

A possible confound in this analysis is that, given a pair of places, it is not possible to exactly match the views and the landmarks visible from the two standpoints. In principle, the classifier could learn to distinguish between two places by learning differences between the distribution of views and/or visible landmarks in the two sets of images. Thus we formally tested whether doing this would increase cross-classification performance. We counted the number of times each landmark was visible in the whole set of images taken from each place, and in the whole set of images depicting each view. This resulted, separately for each place and for each view, in a vector of eight elements (one per landmark) representing the distribution of visible landmarks associated to each place or view. The Euclidean distance between vectors was used as a measure of dissimilarity in the distribution of visible landmarks between a place and a view. For each possible pair of places (e.g., P1 and P3), we checked whether the distribution of landmarks seen from either place (e.g., P1) was more similar to the distribution of landmarks seen when the camera pointed towards the corresponding view (e.g., V1) than towards the other view (e.g., V3). If this was the case, the classifier could in principle exploit this similarity to cross-classify places and views better than chance. It turned out however that the reverse was true, i.e., the distribution of landmarks seen from a given place was significantly more similar to the distribution of landmarks seen when the camera pointed towards other views than towards the corresponding view (*t*_28_ = 7.02, *p* < 0.0001). Thus, learning landmark identities instead of places (or vice versa) would worsen rather than facilitating cross-classification of places and views.

## Results

### Adaptation effects for place, view and heading

Our participants were exposed to a sequence of snapshots taken from a familiar virtual room, each of which could be identified based on the location of the virtual camera (*place*), on the portion of the room that was visible (*view*), and on the camera direction (*heading*). These three spatial features are key “ingredients” of a neural representation of our spatial surroundings, and our analysis was aimed at finding a neural “signature” for each of them, i.e., empirical evidence for a direct neural coding of these features. In particular we looked at a set of predefined regions of interest (ROIs): the PPA, the RSC, the anterior (aHC) and the posterior hippocampus (pHC); but we also performed a complete search across the whole brain.

We adopted two complementary strategies, based on adaptation effects for repeated items and on multivoxel pattern classification, respectively. fMR adaptation, i.e., the reduction in neural activity following stimulus repetition, has been widely used to examine sensitivity to specific visual stimuli and to infer the nature of the underlying representations (Grill-Spector et al., 2006). Here we used fMR adaptation to infer place-, view-, and heading-related representations within the human brain. To do this, we compared three kinds of repeated trials to a common non-repeated condition, by labeling each trial as a function of its relationship with the previous trial (Figure 2A). In same-place (SP), same-view (SV), and same-heading (SH) trials, the place, view, and heading direction, respectively, were the same as in the previous trial, while the other two features differed. In all-different trials, neither the place nor the view nor the heading direction was the same as the previous trial.

We first looked at adaptation effects in the predefined ROIs (Figure 2B). We observed a significant neural attenuation for all kinds of repeated trials as compared to non-repeated trials in PPA (SP: *t*_1,15_ = −4.13, *p* < 0.001; SV: *t*_1,15_ = −5.06, *p* < 0.001; SH: *t*_1,15_ = −4.19, *p* < 0.001; AS: *t*_1,15_ = −6.97, *p* < 0.0001) and RSC (SP: *t*_1,15_ = −4.63, *p* < 0.001; SV: *t*_1,15_ = −5.82, *p* < 0.001; SH: *t*_1,15_ = −3.51, *p* < 0.001; AS: *t*_1,15_ = −6.59, *p* < 0.0001). We did not observe significant neural adaptation in the hippocampus (*p* > 0.01 Bonferroni-corrected).

We then looked for adaptation effects through the whole brain. Regions exhibiting significant response reduction (*p* < 0.01; cluster-level FDR-corrected) when the same place, view or heading were presented in consecutive trials, as compared to no-repeated trials, are shown in Figure 2C. Place-related adaptation (Figure 2C, blue patches) were observed in the lingual/parahippocampal gyrus and in the retrospenial cortex/parieto-occipital sulcus (as already suggested by the ROI analysis), but also in a wide network encompassing the parietal, occipital and frontal lobe. The parietal regions included the posterior part of the intraparietal sulcus (pIPS) and extended more anteriorly into the supramarginal gyrus (SMG) and medially into the precuneus (PCu). The occipital regions bilaterally included the inferior and middle occipital gyri (MOG) and extended medially into the early visual cortex (EVC). The frontal regions included three main foci on the right hemisphere: (i) the superior frontal gyrus (at the intersection with the precentral sulci), which probably corresponds to the human frontal eye fields (FEF); (ii) the inferior frontal gyrus (IFG); and (iii) a portion of the middle cingulum, which corresponds to the rostral portion of the supplementary motor area (pre-SMA). Place-related adaptation effects were also observed in the bilateral thalamus (Tha) and in the inferior temporal gyrus (ITG). Reduced fMRI response in all the aforementioned areas was also observed in the case of same-view trials (Figure 2C, red patches). Significant adaptation effects for repeated heading were observed in a subset of the above-mentioned regions, including bilateral RSC, right PPA, bilateral pre-SMA, pIPS, MOG and right SMG, FEF, ITG and EVC (Figure 2C, green patches).

### Multivariate decoding of place, view, and heading

The second strategy used to reveal neural signatures of place, view, and heading was through multivariate classification analysis. Here we explored whether a linear classifier was able to correctly decode the place, view, or heading direction from multivoxel patterns of estimated neural activity. We obtained separate estimates of neural activity evoked by each of the 32 possible combinations of place, view, and heading, and in separate analyses we grouped the resulting conditions by place, view, and heading (Figure 3A). We trained a linear classifier to distinguish between each possible pair of categories (chance level = 0.5) using a leave-one-out cross-validation scheme. For instance, we tried to decode the specific view visible in each trial from one run from the patterns evoked from all trials of the other runs. Decoding rates for each feature were obtained by averaging the decoding performance across all place/view/heading pairs.

We first analyzed the response in our predefined ROIs (Figure 3B). Decoding accuracy was significantly above chance in all regions of interest (places: aHC mean accuracy = 0.67, *t*_15_ = 20.54, *p* < 0.0001, pHC mean accuracy = 0.63, *t*_15_ = 13.89, *p* < 0.0001, PPA mean accuracy = 0.59, *t*_15_ = 11.63, *p* < 0.0001, RSC mean accuracy = 0.57, *t*_15_ = 10.80, *p* < 0.0001; views: aHC mean accuracy = 0.68, *t*_15_ = 26.35, *p* < 0.0001; pHC mean accuracy = 0.64, *t*_15_ = 24.02, *p* < 0.0001; PPA mean accuracy = 0.62, *t*_15_ = 10.25, *p* < 0.0001; RSC mean accuracy = 0.56, *t*_15_ = 8.13, *p* < 0.0001; heading directions: aHC mean accuracy = 0.66, *t*_15_ = 16.62, *p* < 0.0001; pHC mean accuracy = 62, *t*_15_ = 23.56, *p* < 0.0001; PPA mean accuracy = 59, *t*_15_ = 12.72, *p* < 0.0001; RSC mean accuracy = 0.56, *t*_15_ = 7.29, *p* < 0.0001).

A further control analysis was motivated by the consideration that, given a pair of places, it is not possible to exactly match the views and the landmarks visible from the two standpoints. It cannot be excluded that learning subtle differences in the distribution of views and/or landmarks across images could potentially help the classifier to decode places. For this reason, we ran a further place classification analysis where, for each pair of places, only views that could be visible from both places were included in the analysis, so that views were perfectly matched across place pairs. This implied a reduction of the number of comparisons, because each place could be compared only with places sharing some views (in practice, room corners could be compared with other corners, and room sides with other sides). Classification performance of places resulting from these adjusted comparisons tended to be even higher, and was still significantly above chance in all ROIs (aHC mean accuracy = 0.72, *t*_15_ = 82.80, *p* < 0.0001, pHC mean accuracy = 0.68, *t*_15_ = 125.21, *p* < 0.0001, PPA mean accuracy = 0.65, *t*_15_ = 57.23, *p* < 0.0001, RSC mean accuracy = 0.61, *t*_15_ = 69.26, *p* < 0.0001). Note that similar controls were not possible for the classification of views and heading directions, so we can not exclude that decoding accuracy for views and heading directions depends in part on information about the identity of visible landmarks.

When classification performance for the three features was directly compared in each ROI, there were no differences, except for the PPA (Condition by ROI interaction, *F*_2,30_ = 9.89, *p* < 0.005) in which classification performance was higher for view than for place and heading (*p* < 0.01). It should also be noted that classification performance was higher in the hippocampus than in PPA and RSC, while the previous analysis showed no adaptation effect at all in the hippocampus (see Figure 2B).

We also performed a whole-brain analysis through a “searchlight” procedure (Kriegeskorte et al., 2006) to search for other regions containing enough neural information to allow to decode place, view, and heading (Figure 3C). Outside the territory of PPA and RSC, decoding was successful only in small portions of the bilateral pIPS and in a small region in the right IFG. This was in striking contrast with the strong effect of adaptation widely found in parietal and frontal regions (see Figure 2C).

### Effects of physical distance on neural adaptation

The data presented so far speaks in favor of a neural representation of place, view, and heading in several regions typically associated with topographical orientation and navigation (PPA, RSC, hippocampus), but also (at least when considering adaptation effects) in parieto-frontal regions which are typically associated with egocentric spatial coding (Galati et al., 2010). We further asked whether the three considered features are represented topographically, in map-like representations, with neural activity reflecting real distances between different places, views and heading directions. To this aim, we looked at distance-related effects on both adaptation (Figure 4, left column) and multivoxel responses (Figure 4, right column).

We first looked for adaptation between pairs of images as a function of the spatial differences between them. We tested for a relationship between neural responses in a given trial and distance between the represented place (view, heading) and the place (view, heading) represented in the previous trial. Visual dissimilarities between consecutive images (texture change) and the number of shared landmarks across images were used as covariates to check for any difference in neural adaptation explained by visual similarity and/or landmark identity rather than by spatial similarity. A significant positive linear effect of place change and view change was found in the PPA (place change: *t*_15_ = 2.34, *p* < 0.05; view change *t*_15_ = 4.15, *p* < 0.001) and in the RSC (place change: *t*_15_ = 2.23, *p* < 0.05; view change *t*_15_ = 5.98, *p* < 0.0001), indicating that activity in these regions scaled with the distance in terms of place and view covered between one trial and the next. In addition, we found that activity in RSC was inversely related to the number of shared landmarks between two subsequent trials (*t*_15_ = 2.56, *p* < 0.05), indicating responsiveness to landmark identity. In PPA the effect of the number of shared landmark was close to significance (*t*_15_ = 1.62, *p* = 0.063). No effect was found in the hippocampus. None of the ROIs showed significant effects of either heading or texture change (*p* > 0.01 Bonferroni corrected). Quadratic and cubic terms were not significant in any ROI.

Beyond these regions, whole-brain analysis showed many other regions exhibiting adaptation effects as a function of place- and view-related distance. The whole-brain effect of place change (Figure 4A, left panel) included bilateral parietal (pIPS, PCu) and lateral occipital regions (MOG), and the right ITG. The effect of view change (Figure 4B, left panel) was particularly robust on the ventromedial surface, but was also significant in pIPS, PCu, MOG, and ITG bilaterally, and in the right SMG. As expected, the texture change had a significant impact on the EVC only (Figure 4C, left panel), indicating that activity in EVC reflected low-level visual changes between consecutive images. All found effects were linear, while quadratic and cubic terms were not significant. We found no effect of heading change anywhere at corrected thresholds.

### Cognitive maps reflecting real distances between places and views

A second, independent way to test for the presence of a map-like representation of places, views, and heading directions employed a representational similarity analysis (Kriegeskorte et al., 2008). The idea behind this approach is that a map-like representation implies that the neural representational space shares some similarity with the physical space. If we consider the neural representation (i.e., the multivoxel activity pattern) evoked by two given places (views, headings), the dissimilarity between them should be dependent on the physical distances between the two places (view, headings). In other terms, nearby spatial locations and directions should evoke more similar distributed neural representations.

Thus we explored whether distances between places, views and heading directions, as well as low-level visual differences and landmark dissimilarities between the images, predicted dissimilarities between evoked neural representations, indexed by Euclidean distances between multivoxel activity patterns. Real distances between places significantly predicted neural dissimilarities mainly in aHC (mean beta = 0.73, *t*_15_ = 7.84, *p* < 0.0001) and pHC (mean beta = 0.41, *t*_15_ = 7.03, *p* < 0.0001), but also in PPA (mean beta = 0.37, *t*_15_ = 4.39, *p* < 0.001) and RSC (mean beta = 0.30, *t*_15_ = 2.95, *p* < 0.01). Also real distances between views predicted neural dissimilarities in these regions (aHC: mean beta = 1.23, *t*_15_ = 5.70, *p* < 0.0001; pHC: mean beta = 0.64, *t*_15_ = 4.60, *p* < 0.001; PPA: mean beta = 0.92, *t*_15_ = 5.70, *p* < 0.0001; and RSC: mean beta = 0.50, *t*_15_ = 3.24, *p* < 0.01). Differences in heading direction, and both low level texture and landmark-based differences did not predict neural dissimilarities in any ROI.

A searchlight analysis was finally conducted to test the same hypothesis across the entire brain. Outside the territory of PPA, RSC and HC, a positive relationship between physical distances between places and neural dissimilarities was found in the bilateral insula, in the SMG and in some foci along the medial and lateral frontal lobes (Figure 4A, right panel). A relationship between view-related distances and neural dissimilarities was found in all the above-mentioned regions and in the lateral occipital and ventromedial cortex (Figure 4B, right panel). Again, we found no effect for heading directions and for landmark dissimilarities, while texture differences were associated with neural dissimilarities in early visual areas (Figure 4C, right panel).

### Cross-decoding between places and views

Since all our ROIs showed a significant above-chance classification performance for both places and views, we asked whether neural representation of these two features was somewhat shared or linked. Alternatively, the same region could represent places and views independently, for example in distinct neuronal populations. We reasoned that, if the representation of places and views is somewhat linked, the activation pattern elicited by a picture taken from a given position (place) would share some similarity with the activation pattern elicited by a picture where the camera pointed at the same position (view).

When we trained the classifier to discriminate between places and then tested whether it was able to discriminate between views, we found that the left RSC was the only area showing a significant above-change classification performance (*t*_15_ = 3.28, *p* < 0.01). This result suggests that the RSC contains similar neural representations for places and views and speaks in favor of a unified location code that generalizes across the observer’s and the observed location.

## Discussion

Here we combined fMR adaptation and multivariate analyses to investigate the neural codes that support the encoding of navigationally relevant information, such as place information about where we stand in the environment (place), the portion of the environment we are looking at (view), and our world-centered orientation (heading). To this end, we took advantage of a continuous carry-over sequence of pictures taken from a familiar virtual small-scale environment in which these information were independently manipulated, allowing to disambiguate brain activity related to either of these pieces of allocentric information. One key aspect of our paradigm is that participants were engaged in a familiarity judgment task that was completely independent of the three studied features, so that the specific effects that we describe on brain activation patterns are to be interpreted as the results of the automatic activation of spatial representations.

The most consistent results of our study is that both adaptation and multivariate analyses converge in providing evidence for place-, view- and heading-related representations in the PPA and in the retrosplenial cortex (RSC). In particular, we showed that: (a) the PPA and RSC are able to encode similarities between different pictures of the same landmark/location (view), taken from different standpoints in different heading directions, and this ability does not depend on the low-level visual similarity between different pictures of the same item; (b) the PPA and RSC are able to encode similarities between pictures of different landmarks/locations that are however taken from the same standpoint (place), in different heading directions; and (c) the PPA and RSC are able to encode similarities between pictures which, although representing different landmarks/locations and being taken from different standpoints, share the same allocentric direction (heading).

A key aspect is that, for both places and views (but not for heading directions), there was clear evidence, both from the adaptation and from the multivariate analyses, that spatial representations in PPA and RSC are organized in the form of “cognitive maps”, i.e., that neural representations maintain some similarity with the spatial structure of the environment they represent. The amount of activation elicited by each picture was in fact proportional to the distance (in terms of both place and view) “traveled” from the previous picture; and the dissimilarity between distributed neural representations evoked by two places or views was proportional to their physical distance. This means that PPA and RSC not only encode similarities between pictures associated with the same place or view, but also encode the amount of dissimilarity between different places and views in terms of their physical distance. Or, put in simpler terms, they truly encode the *allocentric spatial location* of places and views. Due to the obvious limitations of the fMRI technique, we cannot speculate about the exact format of the neural encoding of these spatial quantities. Our results are for example compatible with the presence of “place” and “spatial view” cells, whose spatial tuning fields might or might be not arranged in topographical order along the neural tissue (as in a real map). However, spatial representations of places and views could be also distributed across neural populations, not requiring any form of sparse coding.

The role of PPA and RSC in representing different spatial aspects of a visual scene is well documented (see e.g., Epstein, 2008). Previous reports have shown that multivoxel patterns in these regions contain information about scene category and specific landmarks (Morgan et al., 2011; Epstein and Morgan, 2012; Vass and Epstein, 2013). Note that successful decoding of views in the current study could be also interpreted as a neural encoding of the identity of landmarks associated with each view. Since landmarks, by definition, occupy a fixed location in space, it is very difficult to disentangle the representation of their identity from the representation of their location. However distance-related adaptation analyses controlled for the number of shared landmarks between images of nearby views, thus effectively disentangling the contribution of landmark identity and location to the observed effects. Interestingly, we reported that the RSC encodes not only the spatial locations of landmarks but also their identity. The RSC showed an adaptation effect proportional to the number of landmarks within the scene shared with the previous trial. This finding corroborates previous observations (Auger et al., 2012; Auger and Maguire, 2013) reporting that the RSC encodes navigationally salient landmarks and contains information about each permanent landmark in a given view.

One study based on fMR adaptation has already provided evidence for encoding of heading direction in RSC (Baumann and Mattingley, 2010). More recently, Vass and Epstein (2013) have explored spatial representations of “location” and “direction” (equivalent to what here we call “place” and “heading”) while participants were asked to report the compass directions of images taken at different intersections around a familiar city area, by using both fMR adaptation and multivariate analyses. They also explored the encoding of “views”, although, unlike in the current study, views were not fully disentangled from the other two spatial quantities, since each view was always presented from the same location and in the same direction. Critically, in our experiment the same view could be instead presented from different places and while heading in different directions. Another important difference is that Vass and Epstein (2013) used a large-scale environment, which did not allow one spatial location to be visible from the other locations. Instead, in our small virtual environment all locations were potentially visible from any standpoint, so the same spatial locations were used either as “places” or as “views” in different trials.

In Vass and Epstein (2013) results, the PPA is shown to encode places, and the RSC both places and heading directions. However, after controlling for visual similarity of the images, only the encoding of places by the RSC was significant. Both regions also encoded views (but see the caveat above about the different meaning of “view” in their and our experiment). Also, there was no evidence for the relationship between neural pattern dissimilarity and physical distances that we report here. We speculate that the critical aspect to be considered when trying to explain the discrepancy between the results of the two studies is the scale of the spatial environment considered. It is possible that a metric, map-like representation explicitly and precisely encoding distances and directions between spatial locations is easier to build up in small-scale spaces, where spatial locations to be encoded are often simultaneously available during navigation (Wolbers and Wiener, 2014). Different intersections within a city area could instead be encoded as a set of distinct small-scale spaces, and distances and directions *between* these spaces could be represented more loosely and imprecisely.

Our results also suggest some functional differences between PPA and RSC. PPA was equally able to distinguish different places and heading directions, but its decoding performance was higher when pattern classification was applied to predict distinct views. Our findings are broadly consistent with previous studies showing that parahippocampal voxels discriminate between different environments better than between different locations (Hassabis et al., 2009), supporting the idea that the PPA focuses on selective discrimination of different views (Epstein, 2008; Park and Chun, 2009). By contrast, the left RSC was the only region showing evidence of cross-decoding between places and views: the neural patterns evoked by different places afforded classification of responses evoked by different views, suggesting the existence of a neural code that generalizes across actually being in one location and simply viewing that location. Previous studies reported that the RSC plays an important role in integrating route-based spatial information with self-motion cues (Wolbers and Büchel, 2005) and in creating an integrated representation over view change (Park and Chun, 2009), by situating the local scene within the broader spatial environment (Epstein et al., 2007). Moreover, RSC responds preferentially to buildings encountered at decision points along a path, but especially when landmarks are presented in a “in-route” than in “against-route” direction (Schinazi and Epstein, 2010). Thus, the RSC, rather than representing location and view separately, may encode combinations of these two types of information. This may support the existence of a unified allocentric location code, allowing to recognize that the location I am in is the same I was looking at a moment ago. Accordingly with this observation, a recent theoretical framework (Vann et al., 2009) posits that the RSC is a key node for interfacing viewpoint-dependent and viewpoint-independent representations. When this conversion process is disrupted by retrosplenial lesions in humans, severe problems of forming and recalling links between landmarks and place information arise. Patients are unable to derive directional information from landmarks they can recognize (Aguirre and D’Esposito, 1999) and, in some cases, to describe routes through maps of familiar places they can draw (Ino et al., 2007). The ability to combine information about the current and the viewed location is also fundamental for landmark-based wayfinding (Epstein and Vass, 2013). Landmarks may be used to determine the current position within a spatial framework that can potentially extend beyond the current horizon and, again, the candidate brain region for this process is the RSC (Vass and Epstein, 2013).

Although we found the existence of heading-related representations in both PPA and RSC, we found no evidence of effects that scale with the amount of heading displacement. These effects were expected in the RSC, due to the presence of head-direction cells in the rodent (Chen et al., 1994; Taube, 1998). However, head-direction cells are tuned to a specific heading direction but insensitive to any changes of these directions (Taube, 2007). The existence of a visual representation of changing heading has been previously demonstrated only in motion-sensitive visual cortical areas (the posterior part of the ventral intraparietal area and the cingulate sulcus visual area) through stimuli simulating self motion (Furlan et al., 2013). If heading change information is extracted from optic flow, the absence of distance-related effects for heading directions may be explained as due to a lack of directional cues simulating self motion in the current experiment.

Beyond PPA and RSC, we also showed the hippocampus to contain information concerning the current place, view and heading within the environment, which permitted successful decoding by the classifier. Also in the hippocampus, there was a relationship between neural representation similarities and physical distances between places and between views, suggesting a map-like neural representation. The use of multivoxel pattern classification to explore spatial representations in the human hippocampus has gained popularity in recent years (see Chadwick et al., 2012 for a recent review). Hassabis et al. (2009) used this approach for the first time and found that four different locations in a virtual environment could be decoded from the hippocampus activity. Also Rodriguez (2011) found that goal locations during virtual navigation can be decoded from the hippocampus activity. However, in Vass and Epstein (2013) the hippocampus did not distinguish between locations and directions. Morgan et al. (2011) also failed to find evidence of a relationship between neural dissimilarities and real-world distances between landmarks within the hippocampus. Again, the hippocampus may be more responsive to small or newly learned, than to large or highly familiar environments (Smith et al., 2012).

There has been a growing debate on the exact contribution, if any, of the hippocampus in allocentric spatial coding. The importance of classical neurophysiological discoveries such as that of place cells has been questioned on the basis that, for example, many fMRI studies fail to activate the hippocampus in tasks which would be supposed to strongly involve allocentric spatial representations. Here the accuracy in decoding places, view, and heading directions from multivoxel patterns was as high, if not higher, for the hippocampus than for PPA and RSC. However, unlike PPA and RSC, the hippocampus did not show any form of fMR adaptation for repeated places, views, and heading directions. This is not entirely surprising, since fMR effects in the hippocampus have been proven easier to demonstrate with multivariate than univariate approaches (see Pereira et al., 2009; Chadwick et al., 2012; see also the discussion below). Thus, our study points out that the hippocampal activity contains sufficient information to decode the three spatial quantities, and that place and view information are organized in the form of cognitive maps.

The most surprising result of our study came from whole-brain analyses, where we searched for effects outside the predefined set of regions of interest. Adaptation effects to place, view, and heading were found in a unexpectedly large set of regions, in particular in the fronto-parietal cortex (pIPS, SMG, FEF, IFG: Figure 2C); many of these regions also showed an adaptation effect proportional to spatial distances (Figures 4A,B, left column). It is particularly surprising to find such effects in parieto-frontal regions, which are known to be crucial for egocentric spatial representations (Committeri et al., 2004; Sulpizio et al., 2013; see also Galati et al., 2010 for a review). One possible interpretation, which we develop below, is that adaptation effects in parieto-frontal cortex reflect a process of egocentric updating of spatial locations rather than a true allocentric encoding of places and views.

In order to support this interpretation, it should be considered that, leaving aside PPA and RSC, univariate and multivariate analysis produced discrepant results in most of the cerebral cortex. As noted above, the hippocampus did not show any adaptation effect but was significant in most multivariate tests. By contrast, most of the parieto-frontal regions which showed significant adaptation effects did not yield significant results either in multivariate classification (Figure 3C) or in representational similarity analysis (Figures 4A,B, right column). There are many methodological reasons why fMR adaptation and multivariate classification may produce discrepant results, a phenomenon which has been reported before (Drucker and Aguirre, 2009; Epstein and Morgan, 2012). Drucker and Aguirre (2009) suggested adaptation to reflect the tuning of individual (or small populations of) neurons, while multivariate classification would be more sensitive to information distributed at a coarser anatomical scale. Epstein and Morgan (2012) proposed that adaptation would reflect dynamic processes that operate on top of the underlying neural code, to which classification analysis would be instead more sensitive. Here we develop the latter interpretation by underlining one aspect that is rarely considered when comparing the two techniques.

Adaptation effects by design depend on the sequence of trials, i.e., on the relationship between one trial and the next, while classification analyses by design are independent from the trial sequence. When applied to spatial locations within the room, the adaptation analysis labels each trial on the basis of its spatial relationship with the previous one (see Figure 2A), but irrespective of the absolute spatial location which is depicted in that trial. By contrast, classification analysis groups trials on the basis of their absolute spatial location (see Figure 3A), irrespective of the spatial location depicted in the previous trial. Put in other terms, adaptation analysis reveals the effect of “being in the same place as before” vs. “being in a different place”, independent of the specific place where I am and where I was; while classification analysis reveals the effect of “being in this particular place” vs. “being in that particular place”, independent of where I was before. Adaptation analysis considers the recent history of visited places, while classification analysis identifies neural patterns which are consistently associated with one place over time, i.e., neural “signatures” of long-term memory traces.

Thus, apart from considerations about possible differences in the low-level neurophysiological mechanisms underlying the two kinds of effects, the two analyses tap into spatial representations at a different time scale. Parieto-frontal regions, which show distance-related adaptation effects, may be sensitive to temporary *changes* in spatial quantities across consecutive pictures, but show no long-term “signatures” associated with specific spatial locations or directions. In other words, parieto-frontal regions may be able to recognize how much I have traveled from the previous trial, but cannot recognize the current place as the same one I visited some time ago. This interpretation is in line with the general idea of purely egocentric spatial maps in the parieto-frontal cortex (Berthoz, 1997; Kravitz et al., 2011), which get dynamically updated when the subject moves, in order to keep current body-centered information available to the senses in register with a stable viewer-invariant representation of the environment structure, which is available elsewhere in the brain. Available allocentric knowledge gets “injected” into the parieto-frontal cortex during navigation in order to maintain correct representations of egocentric locations of relevant landmarks, some of which may not be visible from the current standpoint. These representations remain however transient, and the updating process alone would not allow the build-up of stable allocentric representations. Such a model (see also Burgess, 2006; Byrne et al., 2007) would predict that activity in parieto-frontal regions scales with traveled distance but does not explicitly encode allocentric spatial locations, which is exactly what we found.

By contrast, the hippocampus exhibited stable neural patterns associated with distinct places, views, and heading directions, and a map-like representation of places, but in the absence of adaptation effects. This speaks in favor of a long-term stable representation of metric spatial relationships, which is scarcely modulated by recent activation history. Along this line of reasoning, the crucial role of PPA and RSC is confirmed by the presence in these regions of both short- and long-term effects resulting from adaptation and classification analyses, respectively.

In conclusion, the current results have important implications for understanding the human system for representing allocentric information. By combining univariate and multivariate analyses, we found that scene-responsive (RSC and PPA), fronto-parietal and lateral occipital regions are automatically recruited while participants view pictures taken from a specific location (place), view and heading within a familiar virtual environment, even in absence of any explicit navigational demand. The hippocampus was identified as crucial in representing place- and view-based distances, the parahippocampal gyrus as specialized in discriminating different views and the retrospenial complex as critical for combining place and view information, while the fronto-parietal cortex showed more transient effects of changes in place, view, and heading. These data support the existence, in the human brain, of map-like spatial representations reflecting metric distances in terms of both one’s own and landmark locations.

## Conflict of interest statement

The authors declare that the research was conducted in the absence of any commercial or financial relationships that could be construed as a potential conflict of interest.
